# *Notes from the Field:* Hurricane Florence–Related Emergency Department Visits — North Carolina, 2018

**DOI:** 10.15585/mmwr.mm6828a3

**Published:** 2019-07-19

**Authors:** Lauren J. Tanz, Molly N. Hoffman, Dana Dandeneau, Zachary Faigen, Zack Moore, Scott Proescholdbell, Susan M. Kansagra

**Affiliations:** ^1^Epidemic Intelligence Service, CDC; ^2^Division of Public Health, North Carolina Department of Health and Human Services, Raleigh, North Carolina; ^3^Council of State and Territorial Epidemiologists, Atlanta, Georgia.

On September 14, 2018, Hurricane Florence made landfall near Wrightsville Beach, North Carolina, as a Category 1 hurricane. Parts of eastern North Carolina experienced 20–30 inches of rain over 80 hours, a record-breaking storm surge of 9 to 13 feet, and maximum sustained wind speeds of approximately 80 miles per hour (*1*,*2*). Surveillance for health outcomes during hurricanes, including emergency department (ED) visits, informs decisions regarding resource allocation and interventions and identifies opportunities to improve emergency preparedness for future disasters.

The North Carolina Disease Event Tracking and Epidemiologic Collection Tool (NC DETECT) is a syndromic surveillance system, collecting data from all 124 civilian EDs in North Carolina. NC DETECT receives data from EDs daily in near real-time on patient demographics, chief complaint, triage notes, diagnosis codes, vital signs, and disposition. NC DETECT was queried to identify Hurricane Florence–related ED visits, defined as ED visits during September 7–28, 2018, resulting from forces of the disaster (e.g., wind and flooding) or direct consequences of these forces (e.g., structural collapse), disruption of normal services, storm preparation or cleanup, stress or anxiety from the storm, or need for shelter. The query was modified from previous hurricane queries in NC DETECT to capture Hurricane Florence–related ED visits with “hurricane,” “Florence,” “flood,” or “storm” in the chief complaint or triage notes.* Record-level data, including patient demographics, chief complaint, and triage notes, were abstracted. Three epidemiologists at the North Carolina Division of Public Health independently reviewed ED visits identified from the keyword query. Visits that indicated the hurricane was a contributing factor and fit the case definition, by reviewer consensus, were considered hurricane-related and were included in the analysis. The reviewers then further classified these hurricane-related ED visits, by consensus, into one of four health categories: injuries, illnesses, medication refills, or other. Percentages of ED visits in each category were compared for periods before (September 7–13), during (September 14–17), and after (September 18–28) Hurricane Florence. Log-binomial models were used to estimate prevalence ratios (PRs) and 95% confidence intervals (CIs) using SAS (version 9.4; SAS Institute).

The hurricane-specific keyword query identified 850 ED visits from 59 EDs; 443 were hurricane-related, including 73 before, 185 during, and 185 after Hurricane Florence. The median age of patients with a hurricane-related ED visit was 50 years (interquartile range = 35–64 years). Among the 73 visits before Hurricane Florence, 25% (18) of hurricane-related ED visits were for injuries, 49% (36) were for illnesses, and 14% (10) were for medication refills (Figure). A similar pattern was observed for hurricane-related ED visits occurring after Hurricane Florence. However, among the 185 visits occurring during Hurricane Florence, 31% (58) of ED visits were for medication refills. Medication refill ED visits were significantly more prevalent during Hurricane Florence, compared with before (PR = 2.3; 95% CI = 1.2–4.2) and after (PR = 2.3; 95% CI = 1.5–3.5), whereas the prevalence of injury and illness ED visits were similar across all periods. After adjustment for age, sex, race, and insurance coverage, medication refill ED visits remained more prevalent during Hurricane Florence, compared with both before and after. Based on descriptions contained in the free text chief complaint and triage notes, disruption of normal services (e.g., closed pharmacies) accounted for 69% (40 of 58) of hurricane-related ED visits for medication refills during the hurricane.

**FIGURE:**
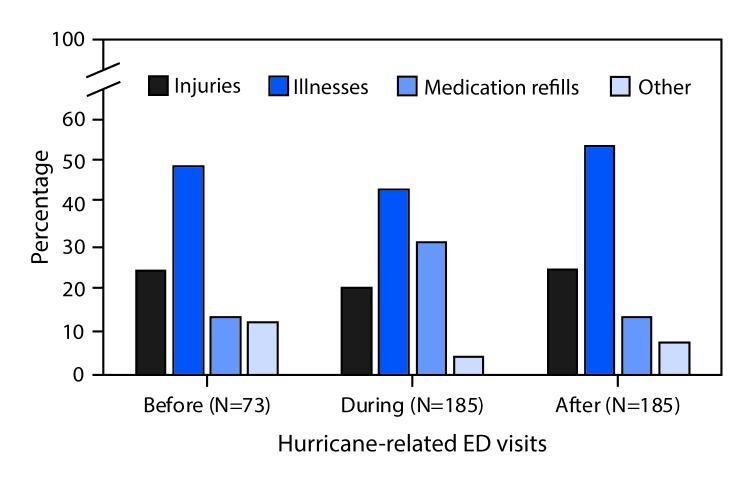
Hurricane-related emergency department (ED) visits before, during, and after* Hurricane Florence — North Carolina, September 7–28, 2018 * Based on dates of service, ED visits were categorized as before (September 7–13); during (September 14–17); or after (September 18–28).

The North Carolina Department of Public Safety disseminated public health messaging regarding emergency kits, evacuation plans, home preparation, and travel safety before, during, and after Hurricane Florence (*3*,*4*). This messaging advised that prescription medicines should be included in home emergency kits. However, during the hurricane, 31% of hurricane-related ED visits were for medication refill. Therefore, it is important that effective messaging to the public, health care providers, and pharmacists before hurricanes emphasize that medications should be refilled to last throughout the storm. North Carolina law permits coverage for extra prescription medication refills during a state of emergency.^†^ Proactive automated pharmacy notifications encouraging patients to refill medications before a potential natural disaster have resulted in small increases in medication refills (*5*). This approach might reduce medication refill ED visits during future natural disasters. In addition, the keyword query used for surveillance of Hurricane Florence–related ED visits in North Carolina could be applied in CDC’s National Syndromic Surveillance Program BioSense Platform and easily modified for use in other states and for other types of natural disasters. This action could enhance natural disaster surveillance nationwide and lead to further query refinement and data analysis that can benefit public health.

## Footnotes

* The keyword query excluded ED visits with “thyroid” to avoid capturing visits related to thyroid storm condition.

† Coverage for extra prescriptions during a state of emergency or disaster. Chap 58 Sect 58-3-228, North Carolina General Statutes, North Carolina General Assembly (2018). https://www.ncleg.net/EnactedLegislation/Statutes/HTML/BySection/Chapter_58/GS_58-3-228.html.
